# Large Bowel Ischemia/Infarction: How to Recognize It and Make Differential Diagnosis? A Review

**DOI:** 10.3390/diagnostics11060998

**Published:** 2021-05-30

**Authors:** Francesca Iacobellis, Donatella Narese, Daniela Berritto, Antonio Brillantino, Marco Di Serafino, Susanna Guerrini, Roberta Grassi, Mariano Scaglione, Maria Antonietta Mazzei, Luigia Romano

**Affiliations:** 1 Department of General and Emergency Radiology, “Antonio Cardarelli” Hospital, Antonio Cardarelli St. 9, 80131 Naples, Italy; marcodiserafino@hotmail.it (M.D.S.); luigia.romano1@gmail.com (L.R.); 2 Department of Radiology, University of Campania “L. Vanvitelli”, Miraglia 2 Sq., 80138 Naples, Italy; donatella.narese@virgilio.it (D.N.); grassiroberta89@gmail.com (R.G.); 3 Department of Radiology, Hospital “Villa Fiorita”, Appia St., km 199,00, 81043 Capua, Italy; berritto.daniela@gmail.com; 4 Department of Emergency Surgery, “Antonio Cardarelli” Hospital, Antonio Cardarelli St. 9, 80131 Naples, Italy; antonio.brillantino@gmail.com; 5 Unit of Diagnostic Imaging, Department of Radiological Sciences, Azienda Ospedaliero-Universitaria Senese, Bracci St. 10, 53100 Siena, Italy; guerrinisus@gmail.com; 6 Italian Society of Medical and Interventional Radiology (SIRM), SIRM Foundation, Via della Signora 2, 20122 Milan, Italy; 7 Department of Radiology, James Cook University Hospital, Marton Road, Middlesbrough TS4 3BW, UK; scaglionefun@gmail.com; 8 Teesside University School of Health and Life Sciences, Middlesbrough TS1 3BX, UK; 9 Department of Radiology, Pineta Grande Hospital, Domitiana St. km 30/00, 81030 Castel Volturno, Italy; 10 Unit of Diagnostic Imaging, Department of Medical, Surgical and Neuro Sciences and of Radiological Sciences, University of Siena, Azienda Ospedaliero-Universitaria Senese, Bracci St. 10, 53100 Siena, Italy; mamazzei@gmail.com

**Keywords:** ischemic colitis, mesenteric ischemia, colon, diagnostic imaging, emergencies

## Abstract

Ischemic colitis represents the most frequent form of intestinal ischemia occurring when there is an acute impairment or chronic reduction in the colonic blood supply, resulting in mucosal ulceration, inflammation, hemorrhage and ischemic necrosis of variable severity. The clinical presentation is variable and nonspecific, so it is often misdiagnosed. The most common etiology is hypoperfusion, almost always associated with generalized atherosclerotic disease. The severity ranges from localized and transient ischemia to transmural necrosis of the bowel wall, becoming a surgical emergency, with significant associated morbidity and mortality. The diagnosis is based on clinical, laboratory suspicion and radiological, endoscopic and histopathological findings. Among the radiological tests, enhanced-CT is the diagnostic investigation of choice. It allows us to make the diagnosis in an appropriate clinical setting, and to define the entity of the ischemia. MR may be adopted in the follow-up in patients with iodine allergy or renal dysfunctions, or younger patients who should avoid radiological exposure. In the majority of cases, supportive therapy is the only required treatment. In this article we review the pathophysiology and the imaging findings of ischemic colitis.

## 1. Introduction

Ischemic colitis (IC), first described by Boley et al. in the 1960s [1], is a pathological condition due to inadequate blood supply to the colon, leading to a different grade of ischemia and potentially causing significant morbidity and mortality [2]. IC is the most frequent form of intestinal ischemia [3] and the second-most common cause of lower gastrointestinal bleeding after colonic diverticulosis [4]. IC is most frequently seen in elderly patients (≥65 years) and increases in incidence with increasing age [5,6,7]; however, it can occur also in younger (≤65 years) patients [8]. The reported incidence is of 16 cases per 100,000 person-years [9], but determining the exact incidence is difficult, as the diagnosis may be easily missed. Indeed, IC may manifest with a mild transient form, with a nonspecific and very variable clinical presentation [10]. The majority of patients complain for diarrhea, hematochezia and crampy abdominal pain [11,12].

## 2. Aetiology and Risk Factors

IC occurs when there is an acute impairment or chronic reduction in the colonic blood supply, resulting in mucosal ulceration, inflammation, hemorrhage and ischemic necrosis of variable severity [13], not always associated with a documented “inflammation”, so ACG suggests the adoption of “colonic ischemia” (CI) as an alternative definition of IC [14].

This condition is in almost all cases related with an impairment of the arterial blood flow rather than of the venous drainage [15,16]. Approximately 15% of patients with colonic ischemia develop intestinal necrosis, with life-threatening consequences, needing prompt diagnosis and treatment. Non-gangrenous ischemia usually resolves without sequelae [17]. The most important risk factor for IC is age, due to the increase in vascular resistance; other significant factors are hypertension, hyperlipidemia, coronary artery disease and diabetes mellitus due to the cardiovascular effects of these conditions. Further are atrial fibrillation, chronic obstructive pulmonary disease, female sex, irritable bowel disease and smoking. Several drugs seems to also be associated with IC in case reports and observational studies: digoxin, diuretics, nonsteroidal anti-inflammatory drugs, cocaine, calcium channel blockers, laxatives, antimotility medications, antihypertensives, neuroepileptics and others [18,19]. Furthermore, IC has been associated with vascular surgery, vascular thromboembolism, hypercoagulable states and all the conditions that may lead to hypotension or hypovolemia [15,20,21,22,23]. In non-elderly patients (≤65 years), hypertension and laxative/enema use were found as risk factors suggesting that colon-related factors might have also a role in non-elderly IC [8].

Two major arteries feed the colon: the superior mesenteric artery (SMA) supplies the ascending and the proximal two/thirds of the transverse colon, and the inferior mesenteric artery (IMA) the distal segment of the transverse colon, descending colon, sigmoid colon and superior segment of the rectum. The internal iliac arteries give blood to the middle and inferior rectum through the middle and inferior rectal artery [24].

There is an anastomotic network that connects the branches of these vessels, constituting the marginal artery, also known as the marginal artery of Drummond. However, there are colonic segments more sensitive to ischemia, particularly the right colon, the splenic flexure (Griffith’s point) and the sigmoid colon (Sudeck’s point), where the marginal artery can be less developed, making these areas more vulnerable to ischemia [23,25,26].

The degree of ischemic vascular impairment, and the damage resulting from it, depend on several factors: the state of the systemic circulation, the anatomical or functional nature of the vascular alteration, the number and size of the affected vessels, the compensatory response of the vascular bed to the reduced perfusion, the nature and capacity of the collateral circulation, the duration of the ischemic insult, the metabolic demand of the intestinal segment involved and the presence/absence of reperfusion phenomena [24,27,28]. Furthermore, the large bowel receives less blood per gram compared to the small bowel, and is more sensitive to autonomic nerve stimulation, which makes it more vulnerable to ischemia [15].

The diminution of arterial blood flow often depends on a decrease in systemic perfusion (e.g., due to dehydration, hypotension or hypovolemia), but can also be due to a local dysregulation of blood supply, or to vessel occlusion. Although it can be associated with several medical and surgical conditions, as listed above, a specific cause cannot be identified in most cases [23,29].

## 3. Clinical Presentation and Classification

Patients with IC usually complain from abdominal pain, rectal bleeding, diarrhea and abdominal distention; nausea and/or vomiting can also be found. Clinical examination usually reveals a soft abdomen. Patients are typically hemodynamically stable, unless presentation with gangrenous IC leads these patients to present abdominal tenderness or signs of frank peritonitis [15]. Laboratory exams may show unspecific alterations [30].

There are multiple classification systems of IC that allow a risk stratification of patients [29,31,32,33]. The simplest divides IC into gangrenous colitis and non-gangrenous colitis. A more detailed system, according to the classification of Brandt and Boley, includes six categories: (1) reversible colopathy (submucosal or intramural hemorrhage); (2) transient colitis; (3) chronic colitis; (4) stricture; (5) gangrene; and (6) fulminant universal colitis [31].

Treatment of IC depends on the severity of presentation. Transient ischemia involving the mucosa and submucosa has a good outcome with conservative management, while fulminant ischemia with transmural infarction leads to a poor prognosis, with a mortality rate reaching 50% [34].

## 4. Investigations

The diagnosis arises from a combination of clinical, laboratory suspicion and radiological, endoscopic and histopathological findings. Sometimes, the clinical presentation is nonspecific or misleading, and it is difficult to distinguish among patients with possible infective, inflammatory, and IC.

### 4.1. Laboratory

Laboratory tests may demonstrate abnormalities depending on the preexisting patient condition, and on the stage of ischemia/necrosis of the bowel wall. Therefore, there are no specific laboratory markers for ischemic colitis. However, inflammatory makers such as C-reactive protein and neutrophil count are likely to be raised. The combination of metabolic acidosis, renal failure and elevated lactate suggest intestinal ischemia and infarction, but these abnormalities are not specific to a diagnosis of IC and are only present in severe and advanced disease. Indeed, for example, a normal lactate value does not exclude full thickness ischemia of the colon [30,35].

### 4.2. Imaging

At present, diagnostic imaging has considerable potential in the diagnosis of and intervention into abdominal pathologies, and was recently also supported by integration with artificial intelligence methods [36,37,38,39,40,41,42,43,44,45,46,47,48,49,50]. The suggestion of ischemic colitis based on imaging findings can permit early diagnosis and prompt therapy, which is essential for a successful outcome [51]. The diagnosis should be confirmed by colonoscopy (CS) and biopsy when possible, even if these procedures are considered unsafe in the acute phase. Guidance from the American College of Gastroenterology (ACG) [52] recommends that computed tomography (CT), the diagnostic investigation of choice, is performed within the first few hours of admission. CS evaluation is recommended within 48 h to detect the bowel alterations and make differential diagnosis [14]. In practice, ultrasound (US) and abdominal X-ray (XR) may sometimes represent the imaging modalities at patient admission for the investigation of abdominal pain, but these imaging methods have no role in directly diagnosing IC [52]. Furthermore, ACG have recognized a role for MRI too; in fact although MRI has been studied in only a small number of patients and, as with CT, such findings could be not specific enough for a definitive diagnosis, MRI may have a role when follow-up imaging is required or in patients with impaired renal function [14,53].

#### 4.2.1. X-ray

XR may be the first imaging technique required for patients complaining of abdominal pain. The imaging findings (highlighted in Table 1) are pale, late, unspecific and non-diagnostic in the majority of cases [54,55].

#### 4.2.2. Ultrasound

According with guidance of the American College of Gastroenterology, there is no role for US in diagnosing ischemic colitis [14]. The limitations of US relate to limited image field-of-view, the operator-dependent quality, and the overlying bowel gas or low-flow vessel disease with a low sensitivity in vessel evaluation [56,57]. However, this investigation is often used in practice in the first assessment of acute abdominal pain. Some authors suggest that it is very sensitive in the diagnosis of colonic mural thickening and in the evaluation of the length of the involved colon, and it can study abnormal echogenicity of pericolic fat and the presence of adjacent peritoneal fluid or free air [58,59,60,61]; it can also give information about blood flow on Doppler US [56,62,63].

#### 4.2.3. CT

##### Imaging Techniques

For a correct evaluation of the bowel, as for a suspected neoplastic or traumatic pathology, abdominal CT should be performed in all patients with a spiral technique and cranio-caudal acquisition, in supine position with abducted upper limbs, thus reducing the radiation dose and guaranteeing a higher image quality of the thoraco-abdominal organs. Acquisitions while breath is held acquisitions ensure avoiding motion artifacts [64,65,66,67,68,69,70,71,72,73].

The examination includes unenhanced and contrast-enhanced acquisitions of the abdomen, with the administration of high-concentration (370–400 mg I/mL) intravenous (IV) contrast medium (CM) (80–130 mL iodinated contrast medium, depending on the patient’s weight), injected at 3.5–4 mL/s through an 18-gauge needle in the antecubital vein, followed by a bolus of 40 mL of saline adopting the same flow rate, to ensure a good vessel depiction. The acquisition of the arterial phase is timed with the bolus tracking placing the region of interest (ROI) on the aortic arch and starting at an attenuation threshold of 100 Hounsfield units (HU). The portal-venous phase is acquired with a delay of 60 to 70 s from the beginning of the injection. The suggested acquisition volume in emergency settings includes chest and abdomen in the portal phase, to obtain a complete examination [51,74,75,76,77]. An additional late scan of the abdomen and pelvis at 3–5 min may be required to determine different causes of abdominal pain [78]; the rectal administration of a contrast material is inutile for IC assessment. For an adequate analysis and post-processing with MIP and MPR, the suggestion is to acquire and reconstruct with thin slice-thicknesses of about 1 mm [79,80]. Automatic tube current modulation should be adopted to reduce radiation exposure and standardize the reconstruction algorithm.

CT acquisitions should be examined with a window setting for the soft tissue (width: 300–350; level: 40–50), to identify changes in the bowel wall, splanchnic organs, mesentery and vessels, and for lung parenchyma (width: 450–100; level: 100–0), to look for wall pneumatosis [81] and extraluminal gas [82,83,84].

##### Imaging Findings

The American College of Radiology (ACR) guidelines recommend CT as the first imaging technique as an alternative to mesenteric angiography for the assessment of IC [85]. CT gives prompt information, with positive findings in ischemic colitis in up to 98% of cases [86,87]. IC is frequently related with non-occlusive etiology, but CT angiography with multiplanar reconstructions along multiple planes is useful to assess the luminal patency of the celiac trunk, SMA, IMA and their main branches, with a high sensitivity and specificity (93.3% and 95.9%, respectively) [88].

In the diagnosis of IC, it is important to indicate [28,89]:

1. the etiology (arterial ischemic, non-occlusive, reperfusive, venous);
2. the location and extension of the intestinal damage;
3. the phase of the damage (acute, subacute or chronic).

1. Etiology

Most cases of ischemic colitis are “low-flow” states and lack the signs of vascular occlusion. Therefore, it is important to focus on the evaluation of the bowel wall instead of vascular occlusive signs.

The type of damage to the bowel wall can be arterial–ischemic or reperfusive.

In arterial–ischemic damage there is an impairment in the colonic blood supply and, if there is no reperfusion, the colonic wall appears thin or “paper thin” in unenhanced and enhanced CT scans. This condition may be observed in patients with non-occlusive mesenteric ischemia (NOMI) (before reperfusion) and in vasculopathic patients with SMA or IMA occlusion, in which the marginal artery is insufficient in providing an adequate blood supply [29]. In addition, lumen dilation and adjacent peritoneal/retroperitoneal fluid may be present, depending on the stage of the ischemia. Wall pneumatosis, pneumoperitoneum, bowel wall hypodensity after IV CM, dilation of the colonic lumen only gas filled (with diameter more than 5 cm), and parenchymal ischemia of the liver, kidney and spleen are unfavorable imaging findings as related with severe hypoperfusion due to bowel infarction with irreversible damage [29,90,91].

**Figure 1:**
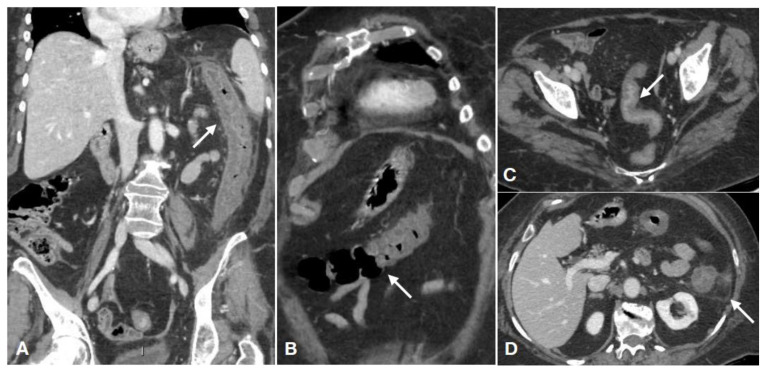
Enhanced CT of a 50-year-old female presenting with abdominal pain and rectorrhagia showing typical CT findings of ischemic colitis. (**A**,**B**) Portal-venous phase in coronal view shows continuous thickening of the colonic wall with mucosal hyperemia, submucosal edema and reduced lumen ((**A**–**D**), arrows). (**B**,**C**) There is involvement of the whole left colon, with a clear distinction between the affected and unaffected segment ((**B**), arrow), and the coexistence of pericolic fat stranding ((**C**), arrow). (**D**) In the axial plane is clearly evident the “little rose” sign ((**D**), arrow). These findings are consistent with ischemic left colitis with reperfusion. No significant vessel findings were detected.

Ischemia may be followed by reperfusion, which is dependent on the hemodynamic support given to the patient and on the effectiveness of the anastomosis circles between IMA and SMA branches (Figure 1). Luckily, in most cases, colonic arterial ischemia is immediately followed by reperfusion through the vascular arcades, and this is the reason for the common appearance of a thickened colonic wall in ischemic colitis (Figure 1) [27,28,29,56,90,92,93,94,95].

**Figure 2:**
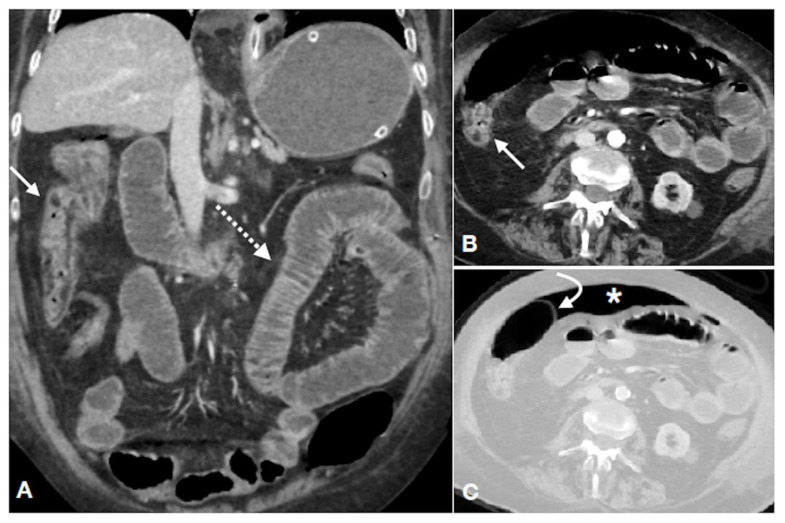
CT of an 85-year-old female with pelvis fracture, complaining of abdominal pain and constipation. Enhanced CT in portal-venous phase, coronal (**A**) and axial (**B**,**C**) views, showing thickening of the ascending colonic wall with mucosal hyperemia and submucosal edema ((**A**,**B**) arrow) and increased enhancement of the small bowel wall ((**A**), dotted arrow); these are findings of small and large bowel reperfusion after ischemia. The colonic wall is partly thickened ((**B**), straight arrow), due to reperfusion phenomena, and partly thinned and distended ((**C**), curved arrow), due to ischemia without reperfusion, leading to colonic perforation with peritoneal free air ((**C**), asterisk).

With reperfusion, various grades of bowel wall injury may occur depending on the time elapsed from ischemia, which consist of vascular congestion of the bowel wall and of the mesentery following restoration of blood flow. This is related with hypoxic damage of endothelial cells and subsequent blood extravasation [27,96]. The reperfused large bowel appears thickened, with mucosal hyperdensity (“little rose” sign) due to hyperemia and hemorrhagic phenomena, with lumen caliber reduction surrounded by peritoneal/retroperitoneal fluid (Figure 1 and Figure 2). The normal thickness of the colonic wall is less than 3 mm [97]; however, it may slight vary depending on the degree of luminal distension [98]. In the absence of distention, the wall is considered thickened if the thickness is more than 1 cm [28]. In more advance stages, a stratified enhanced wall can be produced due to alternating rings of enhancing mucosa with adjacent hypodense edematous submucosa [28]. If reperfusion does not occur after the acute impairment of the colonic blood supply, the colonic wall appears “paper thin” (Figure 2).

The venous etiology of ischemic damage is rare and depends on the impaired blood outflow with bowel wall thickening and congestion, very similar to the reperfusion damage from which it may be distinguished by the presence of superior or inferior mesenteric vein obstruction or the presence of portal venous system congestion [16,74,99].

Recent evidence supports the use of dual energy CT, adopting iodine maps and low monoenergetic images to improve the evaluation of bowel wall enhancement and, particularly, to evaluate the effectiveness of the reperfusion and the related, more appropriate treatment [100,101,102,103,104].

2. Location and extension of the damage

CT is also useful to assess the colonic involvement:

A segmental involvement is usually seen in occlusive IC. This is explained by the vascular anatomy, while in the case of non-occlusive damage, no regions are spared from involvement (Figure 1);

Multisegmental involvement is more frequently observed than single-segment involvement;

Contiguous multisegmental involvement is more frequent than skipped segments involvement;

The most frequently involved segment is the descending colon, followed by sigmoid colon, right colon and the entire colon [105];

Isolated right colonic ischemia (ischemic changes proximal to the hepatic flexure) has a higher frequency of surgical intervention and a higher mortality when compared with non-isolated right colonic ischemia [14,15].

3. Phase of the damage (acute, subacute or chronic)

The phases of the damage can be acute, subacute or chronic.

In the acute phase, it is important to differentiate ischemic (occlusive and non-occlusive) and reperfusion damage, whereas in the subacute phase it is crucial to monitor the status of reperfusion: the thickening of the wall and adjacent peritoneal/retroperitoneal fluid should progressively decrease with progressive restoration of the physiological wall thickness. In the chronic phase, it is possible to identify ischemic colonic stricture, due to fibrosis of the bowel wall with loss of the haustral fold.

CT imaging findings are summarized in Table 2.

#### 4.2.4. MRI

##### Imaging Technique

In recent years, there has been increasing use of MRI imaging for the study of bowel and gastrointestinal pathology [39,106,107,108,109,110,111,112]. From the available studies, it has emerged that MRI may allow for the identification of pathological findings of acute IC and their correlation with histopathological features [28,29,90,113]. Particularly, the advantage of the adoption of MRI for the clinical management of IC is the possibility of performing a short-term follow-up without the employment of ionizing radiation or intravenous contrast material [53,114,115].

**Figure 3:**
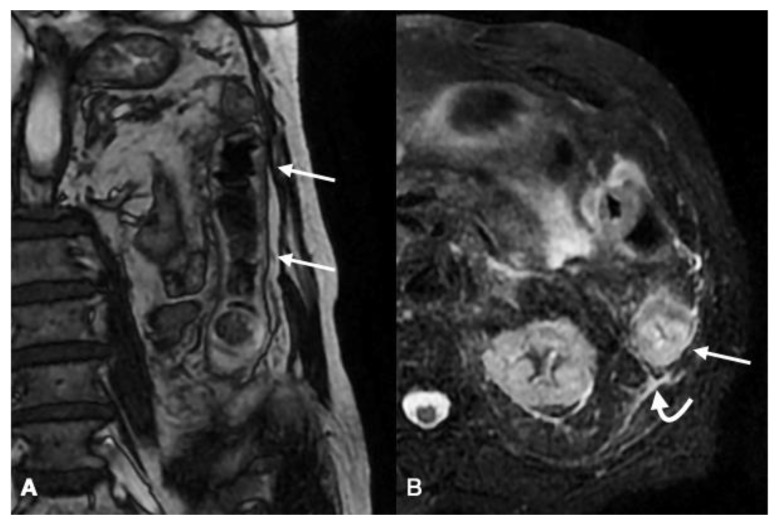
Follow-up MRI in an 80-year-old female with ischemic colitis, (**A**) T2W FIESTA sequence on the coronal plane and (**B**) T2W FatSat sequence on axial plane showing the continuous involvement of the descending colon ((**A**), arrows), characterized by oedematous colonic wall thickening ((**B**), arrow) with pericolic fluid ((**B**), curved arrow).

Performing MRI with a 1.5 T magnet has been suggested, adopting a surface coil with T2-W multiplanar sequences, as in T2W sequences we can clearly detect the wall edema and pericolic fluid, which are the main features of ischemia with reperfusion [29,53] (Figure 3). Oral and intravenous medium contrast are usually not administered. Without the intravenous contrast medium injection, the evolution of changes in the appearance of the colonic wall may be appreciated, but vascular findings cannot be evaluated.

##### Imaging Findings

T2W sequences allow us to detect the hyperintense signals of the bowel wall, which are mainly superficial in mild ischemic–reperfusion damage, and more pronounced and extended into the submucosal layer in more advanced stages of reperfusive damage. In addition, a variable amount of pericolic fat stranding and peritoneal/retroperitoneal free fluid may be detected [19,28,29,113] (Figure 3).

The IV CM is useful for evaluating the bowel wall vascularity; however, its use may be limited to selected cases and doubtful findings from T2W sequences.

The main technical limits of MRI are related with patient compliance, the presence of MR-unsafe implanted devices, and machine availability. Regarding the imaging findings, it may be easier to miss pneumatosis and free air when compared with CT. However, there are also other findings (bowel wall thickness and its signal) that have be considered in order to evaluate the patient’s evolution.

#### 4.2.5. Mesenteric Angiography

Before technical advances in CT angiography, mesenteric angiography (MA) represented the gold standard for diagnosis and treatment. By now, due to the availability of less invasive imaging methods such as CT and MR, its role is reserved for specific therapy of stenosis/occlusion of the arterial mesenteric vessels, or the treatment of venous thrombosis.

## 5. Differential Diagnosis

The described imaging findings of ischemic colitis are suggestive, but not pathognomonic, for the diagnosis, and so a panel of different conditions should be considered, mainly represented by infectious colitis, diverticulitis, appendicitis, typhlitis, graft-versus-host disease (GVHD), radiation colitis and inflammatory bowel diseases (IBD) [67,107].

To reach the correct diagnosis, emphasis should be placed on the patient’s clinical history, on the geographic distribution of the abnormalities, and on the presence of extra-intestinal findings. The patient should be asked about previous pathologies, surgeries and drugs consumption, and about the timing of onset of the acute symptoms and their characteristics.

The presence of vessel findings such as arterial filling defects, aortic dissection with the involvement of mesenteric arteries, small arterial aneurism and occlusion (in the setting of vasculitides), and venous thrombosis may help in the diagnosis of ischemic bowel, as will the presence of signs of ischemic damage involving other visceral organs (i.e., the liver, kidneys, spleen and pancreas) (NOMI) [116].

The clear demarcation of ischemic and non-ischemic tracts is typical of occlusive ischemic conditions [29], even if in NOMI the colonic involvement may vary based on the susceptibility of the bowel wall to ischemia.

Typhlitis is typically localized in the cecal area, while in acute appendicitis we may see a variable inflammatory involvement of the adjacent bowel [117], in radiation colitis there is a segmental involvement of the irradiated bowel, and in infectious colitis the extent of involvement may vary and can manifest as pancolitis. Bowel involvement is concentric and continuous in distribution in ulcerative colitis, whereas in the majority of cases eccentricity, segmental distribution and greater prominence are found in Crohn Disease [67,118].

Upon finding colonic thickening, its characteristics should be analyzed.

A strong mucosal enhancement with “accordion sign” is more suggestive of pseudomembranous colitis, whereas irregularity in the mucosal layer with ulcers is suggestive for IBD; a slight mucosal hyperemia may be found in other conditions [67,118]. The stratified pattern of the wall can be found in multiple inflammatory conditions, as well as the “fat halo” and “water halo” signs. The appearance of perivisceral fat may suggest ischemic and infectious conditions in the presence of fat stranding and pericolic fluid, whereas we are directed towards IBD in cases of fat hypertrophy, a “comb sign” or the presence of fistulas. Attention should also be payed to pneumatosis, which is not always a synonym of wall necrosis, but is related with infection or lumen overdistention [81,92,119,120,121].

Therefore, diagnostic imaging may suggest the final diagnosis, however it needs to be contextualized to a multidisciplinary approach taking into account the medical history, clinical and endoscopic signs, anatomical-pathological findings, and the patient’s laboratory tests.

## 6. Colonoscopy

Colonoscopy is considered the test of choice in confirming the diagnosis of IC, due to its high sensitivity. An early colonoscopy is suggested by ACG guidelines within 48 h of IC presentation, with minimal insufflations and stopping at the most distal extent of the disease [14]. It is able to demonstrate mucosal damage/changes and allow the performance of biopsies [122].

## 7. Treatment Possibilities

According to the ACG clinical guidelines [14], IC treatment should be based on disease severity [14,123,124,125]. In patients with mild symptoms of IC, treatment is basically supportive (intravenous fluids injection and bowel rest) and observational. Surgical intervention should be considered in patients with moderate or severe IC, and if there is imaging evidence of advanced ischemia and perforation, generalized peritonitis, or bleeding causing instability or repeated transfusion. In the absence of these specific elements, the decision between operative and conservative management is made on clinical basis. Operative intervention usually includes resection and colostomy.

## 8. Conclusions

IC diagnosis is strictly related to clinical suspicion/manifestation and clinical history, especially when other conditions are superimposed (e.g., infectious colitis, inflammatory bowel disease, diverticulitis, colon cancer).

More frequently, IC can presented with abdominal pain, diarrhea and hematochezia, or remain silent.

Even if endoscopy is considered the diagnostic test of choice in diagnosing IC, imaging tests are usually performed before. US and XR use is limited because they yield non-specific and late findings. CT examination is the technique of choice for the noninvasive diagnosis of IC, and to make differential diagnoses between other causes of acute abdominal pain. However, it requires the use of ionizing radiation and an iodinated contrast agent, limiting the possibility of repeatedly using this technique in a short-term follow-up; in this sense, MRI may be useful.

## Figures and Tables

**Table 1:** Imaging findings from plain radiography of the abdomen in patients with IC.

Gasless Abdomen	Paucity of Intestinal Gas Due to Spastic Reflex Ileus
Large bowel dilation	Distention of the colonic lumen, similar to that seen with toxic megacolon inflammatory bowel disease may occur in fulminant ischemic colitis.
Bowel distension proximal to the involved colon	
“Colonic thumbprinting”	The most specific finding of colonic ischemia. It appears as loss of haustration and dilation of the colon with rounded densities along the colon wall. These multiple round, smooth soft-tissue densities projecting into the air-filled colonic lumen are caused by submucosal edema or hemorrhage.
Rigidity, narrowing and lack of haustrations and a tubular appearance of the bowel	May develop as the edema progresses.
Hepatic portal venous gas Intestinal pneumatosis Free air in the peritoneal cavity	Associated with bowel necrosis. These are signs of advanced ischemia that should be further investigated with CT examination

**Table 2:** Abdominal CT imaging finding in patients with IC.

Damage	Radiological Findings and Characteristics
Type of damage Arterial ischemic type (less frequent) Reperfusive type (more frequent) Venous congestion (rare)	Thin or “paper thin” colonic wall;
	Unenhanced colonic wall at enhanced CT;
	Dilation of the lumen, only gas-filled;
	Wall pneumatosis;
	Pneumoperitoneum;
	Parenchymal ischemia of liver/kidney/spleen;
	SMA/IMA or relative branches obstruction;
	Peritoneal/retroperitoneal free fluid (late finding);
	Thickened colonic wall;
	Mucosal hyperdensity (“little rose” sign);
	Lumen caliber reduction;
	Stratified enhanced wall (“target sign”);
	Fat stranding;
	SMA/IMA or relative branches obstruction;
	Pericolic fluid;
	SMV/IMV or relative branches obstruction;
	Bowel wall findings similar to the reperfusive type;
	Peritoneal/retroperitoneal free fluid
Location of the damage	Descending colon;
	Sigmoid colon;
	Right colon;
	Entire colon
Extension of the damage	Single segment involvement;
	Multisegmental pattern:
	Contiguous multisegmental involvement
	Skipped segments involvement
Phases of the damage	Acute
	Subacute
	Chronic

## Data Availability

Data sharing not applicable.
